# Techno-Economic Analysis of Membrane-Based Plants for H_2_/CH_4_ Purification

**DOI:** 10.3390/membranes15110336

**Published:** 2025-11-07

**Authors:** Pasquale Francesco Zito

**Affiliations:** National Research Council—Institute on Membrane Technology “Enrico Drioli” (CNR-ITM), Via P. Bucci, Cubo 17 C, 87036 Rende, CS, Italy; p.zito@itm.cnr.it

**Keywords:** H_2_ purification, CH_4_ upgrading, membrane separation, economic analysis

## Abstract

In the context of the growing adoption of alternative gas separation processes, combined with the interest in hydrogen as a fuel and energy carrier, the use of membrane technology in H_2_/CH_4_ purification is analyzed in this work, focusing on the techno-economic aspects. In particular, the separation and economic performance of three Pd–Ag/Si-CHA membrane plants are simulated, aiming to achieve high degrees of purity and recovery paired with cost-effective configurations. A single Pd–Ag membrane stage operating at 20 atm and 350 °C can theoretically guarantee a CH_4_ concentration of 95%, while a completely pure H_2_ stream leaves the plant as a permeate product. The choice of a less selective Si-CHA membrane allows a temperature reduction but implies the use of more stages to achieve the desired CH_4_ target. In addition, H_2_ purity does not exceed 98%. A two-stage hybrid process, in which the retentate gas leaving the Pd–Ag membrane is cooled and fed to the Si-CHA unit, is also a cost-effective solution, as feed pressure can be reduced to 10 atm with significant compression cost savings. All the configurations are able to provide positive values of economic potential (EP); however, the single Pd–Ag membrane plant is the best option since it guarantees the highest EP, net profit and net present value (NPV).

## 1. Introduction

In the gradual transition from fossil fuels to renewable energy, decarbonization is a crucial step, especially to achieve the desired target of net-zero emissions by 2050 [1]. In this context, hydrogen represents a key component, since its combustion does not produce carbon dioxide but only water. Global hydrogen demand exceeded 97 Mt in 2023, approaching 100 Mt in 2024 [2,3]. Nevertheless, its adoption in energy transition still accounts for less than 1% of the demand, whereas the main application fields are still refining and industry [2].

The Fuel Cell and Hydrogen Energy Association (FCHEA) splits hydrogen consumption in the US hydrogen market as follows: 57% in oil refining and 38% in ammonia and methanol production, with the remaining percentages for metals and other applications [4]. A further expansion in ammonia production is expected in the years to come. Global hydrogen production occurs especially from natural gas (about 48%), oil (30%) and coal (18%); only 4% occurs by water electrolysis [5]. Based on this data, hydrogen is obtained especially by methane steam reforming, autothermal reforming or natural gas decarbonization [6]. On the other hand, the Chinese hydrogen production market is dominated by the use of coal; in fact, it accounts for about 62%, followed by gas, whereas water electrolysis accounts for 1% [7]. The main limitation of water electrolysis is the high cost compared to the other technologies. However, the European Union industry has the ambitious plan to install 2 × 40 GW of H_2_ electrolyzers by 2030 in Europe and its neighborhoods [8].

In the hydrogen production processes from mixtures such as natural gas, coke oven gas and ethylene/refinery tails, H_2_ and CH_4_ are the main components, accounting for more than 95% [9]; hence, H_2_/CH_4_ separation is of great industrial interest. A valuable component can be obtained for several applications, depending on the degree of purity. A high H_2_ quality (i.e., 99.97% purity or higher) is required in transportation to power PEM fuel cells; a lower degree of purity (>98%) is accepted for internal combustion engines, direct injection in the gas grid and domestic uses [10]. Hydrogen can be safely stored in liquid carriers (e.g., organic as methanol and dimethyl ether, or inorganic as ammonia, etc.), making this a cost-effective alternative to gas compression and low temperature liquefaction [11].

Various methods can be used for purification of H_2_ from CH_4_ or other permanent gases, such as pressure swing adsorption, chemical absorption and cryogenic distillation, requiring complex equipment and high operating costs [12]. PSA is the most widespread method to purify H_2_ (about 99.999%) from a multicomponent mixture, accounting for about 85% [10]. However, in recent decades, the attention has focused on membrane processes for the separation of gas mixtures, exploiting their environmental sustainability, compactness, simplicity, flexibility, low energy requirement, etc. [13].

H_2_ purification is generally carried out using Pd membranes, which are able to guarantee an ultrapure component due to their theoretically unlimited H_2_/gas selectivity [14]. Nevertheless, some issues still present limit their use at an industrial scale: membrane embrittlement at a low temperature, high cost and inhibition effect in the presence of other gases (especially CO) [13,15,16]. Some solutions to mitigate these negative effects are given by using Pd–alloy membranes, such as Pd–Ag and Pd–Cu.

Alternative solutions for H_2_/CH_4_ separation are given by different organic and inorganic materials, such as polymer, zeolites and graphene, even if these materials are not able to achieve the H_2_ purity levels of a Pd membrane. Polymer membranes are the most investigated in this field due to several reasons, such as easy synthesis process, good mechanical strength, low cost and scalability [17]. Their main limitations are represented by the permeability/selectivity trade-off paired with some undesired phenomena, such as plasticization and swelling, that drastically negatively affect the separation performance [18]. Zeolite membranes are a possible alternative, being able to guarantee a high chemical and thermal stability paired with high flux and selectivity, even if some important drawbacks are present, such as the higher investment costs, difficulty in reproducibility and scalability, etc. [19]. To face the problem related to expensive synthesis, research efforts are moving towards novel green synthesis methods to make the preparation process cheaper and environmentally sustainable [19].

Several kinds of zeolites are able to separate H_2_ from CH_4_. Table 1 shows some mixture separation performances of various small-pore zeolite membranes at ambient temperature and different feed pressures.

**Table 1:** H_2_/CH_4_ separation performance of some zeolite membranes.

Zeolite-Type	Temperature, Feed Pressure and Composition	H_2_ Permeance, mol m^−2^ s^−1^ Pa^−1^	H_2_/CH_4_ Selectivity, -	Ref.
Si-CHA	25 °C, 3 bar, equimolar	14.4 × 10^−7^	85	[20]
	25 °C, 11 bar, equimolar	≈9.0 × 10^−7^	≈45	
SAPO-34	25 °C, 3 bar, equimolar	14.5 × 10^−7^	42.2	[21]
	25 °C, 13 bar, equimolar	≈7.0 × 10^−7^	≈18	
SSZ-13	25 °C, 3 bar, equimolar	1.8 × 10^−7^	43	[22]
	25 °C, 11 bar, equimolar	≈1.2 × 10^−7^	17	
AIPO-18	25 °C, 3 bar, equimolar	1.0 × 10^−7^	22	[23]
	25 °C, 11 bar, equimolar	≈0.6 × 10^−7^	≈18	

This work aims to investigate the potential of using a membrane-based process for the purification of H_2_ and CH_4_ from their binary mixture, simulating various large-scale plant configurations. The novelty lies in combining zeolite and Pd-based membranes at an industrial scale to simultaneously purify CH_4_ and H_2_ from their mixture, while evaluating the economic feasibility of the proposed process. The separation performance of the proposed three schemes using Pd–Ag and zeolite membranes is evaluated and compared to identify the solution able to provide the best results in terms of recovery and purity. An economic assessment is carried out to discover which solution is the most cost-effective and profitable, estimating the economic potential (EP), net profit and net present value as functions of the raw material price.

## 2. Materials and Methods

The performance of the simulated plants is based on the solution of the mass balances for the two species, which allows the evaluation of compositions and flow rates. For a generic component *i*, Equation (1) describes the steady state mass balance along the infinitesimal membrane length *dz*; when the mass balance is referred to a Pd-based membrane, Equation (2) is generally used for describing the H_2_ permeation [24]:

(1) $$-\frac{{dF}_{i}}{dz}={Pe}_{i}\pi D\left(P_{i\left(z\right),\;Ret}-P_{i\left(z\right),\;Perm}\right)$$

(2) $$-\frac{{dF}_{H_{2}}}{dz}={Pe}_{H_{2}}\pi D\left(P_{H_{2}(z),Ret}^{0.5}-P_{H_{2}\left(z\right),\;Perm}^{0.5}\right)$$

The ordinary differential equations are solved with the following model assumptions:

- No pressure drops along the membrane modules;
- Validity of Dalton’s law;
- H_2_ permeation through Pd–Ag membrane described by Sievert’s law (Equation (2));
- H_2_/CH_4_ infinite selectivity through Pd–Ag membranes;
- Constant permeance and selectivity values along the Si-CHA membranes.

The validity of Equation (1) with the related assumptions has been demonstrated by comparing its predictions with some laboratory experiments on different types of membranes [25,26,27]. In addition, a comparison with other simulation results of Scholz et al. [28] has been carried out in a recent publication [29]. Regarding the gas permeation through the Pd-based membrane, simulations are validated against experimental results, expressed as H_2_ recovery for a hydrogen/helium mixture permeating through an area of 678 cm^2^ [30]. A recovery of 99.91% is estimated here by simulations, which is close to the 97.74% reported in the literature [30].

Input data for simulations are summarized in Table 2. Feed flow rate is set to 100,000 mol/h (i.e., 2240 Nm^3^/h), considering small-size plants (i.e., <6000 Nm^3^/h [31]). Concerning the Pd–Ag membrane, H_2_ permeance is estimated from its experimental trend as a function of temperature investigated by Brunetti et al. [32]. In fact, the mathematical expression allows estimating the value at 350 °C, which is 1.9 × 10^−3^ mol m^−2^ s^−1^ Pa^−0.5^. Similar permeances on the order of 10^−3^ mol m^−2^ s^−1^ Pa^−1^ have been found recently in the literature at 400 °C [33,34,35]. In 2025, Jazani et al. [33] estimated a permeability of 7.5 × 10^−8^ mol m^−1^ s^−1^ Pa^−0.5^, referring to a Pd–Ag–Y membrane having a thickness of 38 μm. This value corresponds to a permeance of about 2 × 10^−3^ mol m^−2^ s^−1^ Pa^−0.5^, in agreement with the value supposed in this work. Similarly, in 2024, Alique et al. [34,35] obtained permeances between 5.37 × 10^−4^ and 1.03 × 10^−3^ mol m^−2^ s^−1^ Pa^−0.5^ at 400 °C in composite Pd membranes. Omidifar and Babaluo [36] fabricated Pd–Ni membranes able to provide infinite H_2_/N_2_ ideal selectivity at 450 °C. From their measurements of flux versus driving force at 400 and 450 °C, a permeance of about 6 × 10^−5^ mol m^−2^ s^−1^ Pa^−0.5^ at 350 °C is extrapolated, being approximately 30 times smaller than the assumed value here (i.e., 1.9 × 10^−3^ mol m^−2^ s^−1^ Pa^−0.5^). This would imply a proportional increase in the membrane area, leading to higher costs. Separation performance of a Si-CHA membrane is taken from the experimental data of Wu et al. [20], which are evaluated at 11 bar. In this work, it is assumed that an increment of pressure from 11 to 20 atm does not affect the permeation of the two components. In fact, various experimental works showed that the most variation is observed in the low-pressure range [20,21,22]. This potential further reduction in permeance would lead to a proportional increase in the zeolite membrane area necessary to maintain the same separation performance. However, this possibility would concern only the second configuration, since the third one operates at 10 bar. In addition, it is assumed that scaling up to larger membrane modules does not alter the permeance values. Purification targets are summarized in Table 3. In particular, a CH_4_ purity of 95% is set to guarantee the minimum biomethane purity from biogas [37]; H_2_ purity is fixed to 98% to have a product able to be used for internal combustion engines, direct injection in the gas grid and domestic uses [10]. Concerning the recovery targets, here, 90% is considered a reasonable value to limit H_2_ losses.

A preliminary economic evaluation is carried out, estimating the economic potential with the following equation (Equation (3)) [38]:

EP = REVENUES − RAW MATERIALS − ONSITE − UTLITIES(3)

The economic parameters necessary in Equation (3) are reported in Table 4. The equations used for the economic analysis are summarized in Appendix A (Equations (A1)–(A5)) and discussed in more detail in a previous publication [29]. Regarding the membrane installation costs, a factor of about 2.5 with respect to the purchase costs is assumed, which are set to 5700 and 1000 $/m^2^ for Pd–Ag and zeolite, respectively [29].

## 3. Results and Discussion

### 3.1. Purification by Pd–Ag Membrane Plant

**Figure 1:**
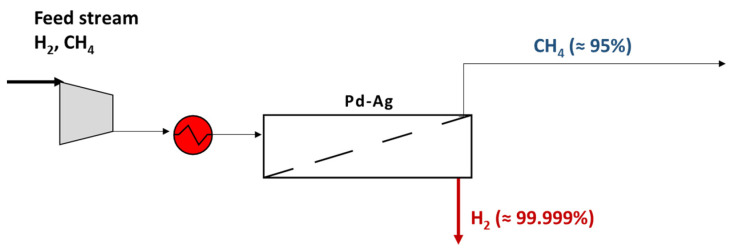
Scheme of Pd–Ag membrane plant for H_2_/CH_4_ purification.

A simple way to carry out the separation is to install a Pd-based unit, in order to obtain pure H_2_ on the permeate side, exploiting its unlimited H_2_/gas selectivity (Figure 1). A preliminary heating of the compressed feed stream is required to avoid membrane embrittlement. In addition, an appropriate feed pressure has to be set to increase the H_2_ driving force, allowing a good permeate recovery and, therefore, an increment of CH_4_ purity on the retentate side too.

**Figure 2:**
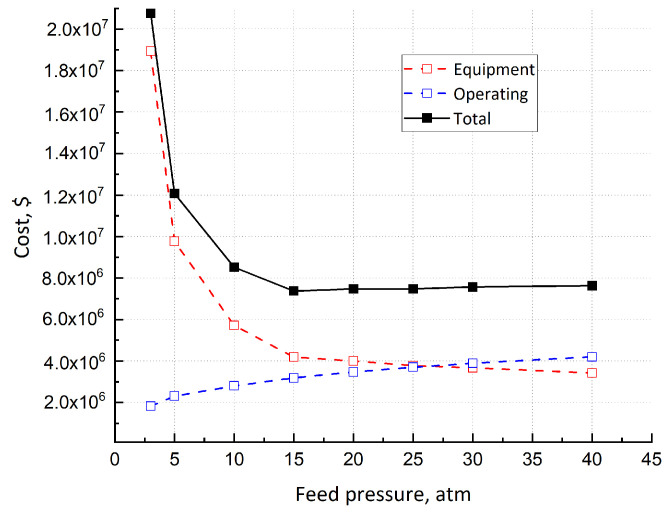
Equipment cost, utility cost and their sum as functions of feed pressure.

**Figure 3:**
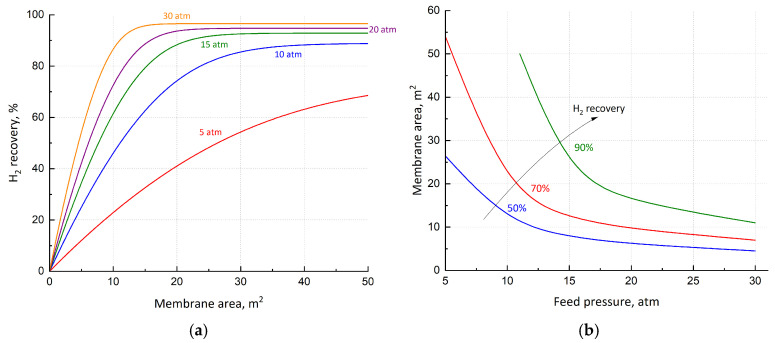
(**a**) H_2_ permeate recovery as a function of feed membrane pressure; (**b**) membrane area as a function of feed pressure at different H_2_ recovery.

The choice of the appropriate feed pressure is based on its effect on several variables, such as H_2_ recovery, CH_4_ purity, Pd–Ag area, installation and operating equipment cost. In terms of separation performance, a higher pressure allows better CH_4_ purification and H_2_ recovery, even though it implies a higher compression cost. The trend of the total cost (operating plus equipment) as a function of feed pressure is depicted in Figure 2. A minimum is observed, due to the balance between the more power for compression and less membrane area, occurring when feed pressure increases. This minimum cost corresponds to a pressure of about 15 atm. At this point, equipment and utility, as parts of CAPEX and OPEX, respectively, have almost the same weight; for lower pressure, CAPEX prevails. It is important to point out that raw material is not considered in this cost analysis, since its price is a variable. However, these pressure values are not able to respect the CH_4_ level of purity. In fact, the minimum required pressure able to theoretically guarantee a CH_4_ retentate concentration of 95% is 20 atm. Therefore, this value is set for the analysis of the single-stage configuration. Higher values of feed pressure do not allow significant improvements in H_2_ recovery (Figure 3a). The direct relationship between membrane area and feed pressure is depicted in Figure 3b. A sharp reduction is observed of up to 10–15 atm, depending on the recovery set. For higher pressures, the dependence is less pronounced, and the membrane area approaches a constant value.

**Figure 4:**
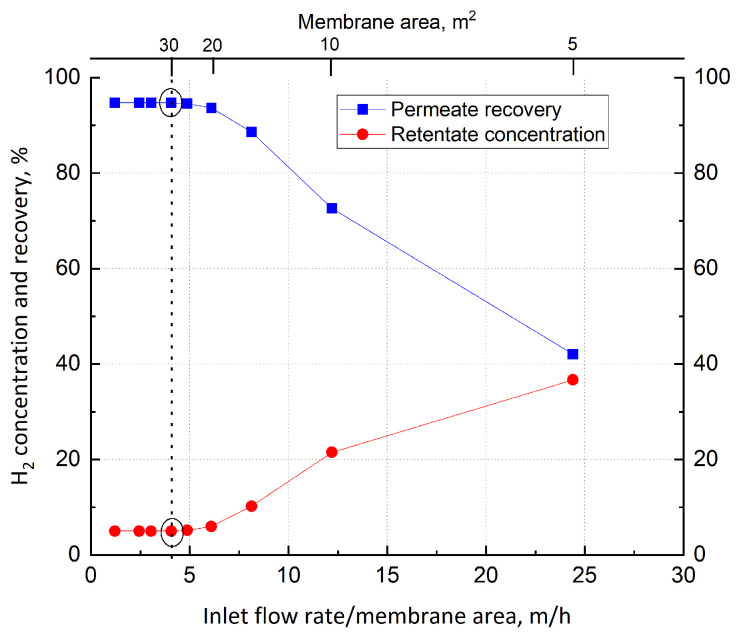
Dependence of H_2_ retentate concentration and permeate recovery on inlet flow rate/membrane area ratio in the case of a single-stage Pd–Ag membrane.

Once the operating pressure is set, the membrane area is selected by analyzing its effect on the purification performance, as shown in Figure 4. However, to make this analysis independent of feed flow rate, the ratio between the inlet flow rate and the area is introduced. A specular behavior between retentate concentration and permeate recovery is observed: above 5 m/h, an increment of this ratio reduces the recovery while increasing concentration, indicating that membrane area is inadequate for the complete separation process; on the other hand, for values lower than 5 m/h, both the parameters are constant, suggesting that the maximum separation has been achieved. Here, a ratio of 4 m/h is set, corresponding to 30 m^2^ in the case of 100,000 mol/h fed. The required area can be achieved by installing several small modules in parallel. This configuration provides a retentate stream containing 5% of H_2_, with a permeate recovery of 94.7%.

**Figure 5:**
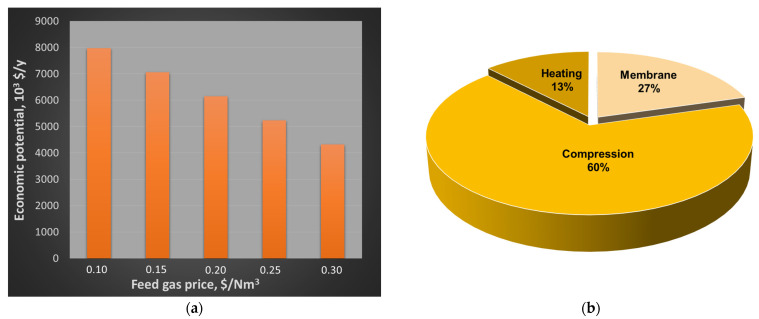
(**a**) EP as a function of the raw material price; (**b**) cost distribution when the raw material is excluded.

The economic analysis, expressed in terms of EP as a function of the feed price, reveals that this configuration can generate profit in a certain interval of raw material price, since EP is positive up to about 0.53 $/Nm^3^ (Figure 5a). In the entire range of prices considered, raw material is the main expense. However, if it was excluded, compression would be the most important cost, accounting for about 60% of the total (Figure 5b). The second cost is due to membrane installation, which is calculated to be more than two times the heating cost (heat exchanger + utility). This occurs because a significant part of the heating cost is recovered assuming that the compressed steam used to achieve the desired temperature of 350 °C leaves the heat exchanger in saturated conditions, being potentially sold as a utility [29,44].

### 3.2. Purification by Si-CHA Membrane Plant

**Figure 6:**
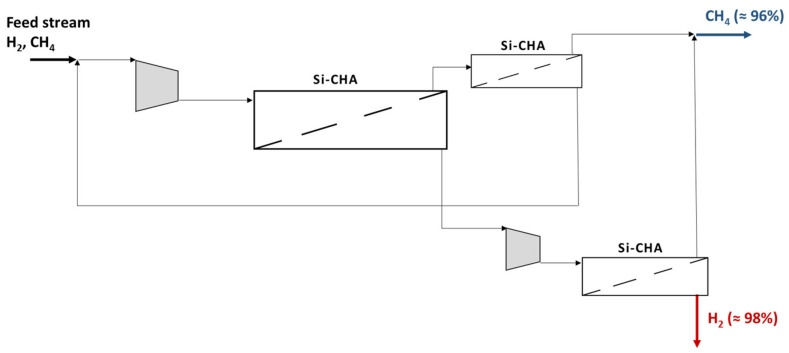
Scheme of the Si-CHA membrane plant for H_2_/CH_4_ purification.

An alternative way to purify H_2_ from CH_4_ is the use of other inorganic membranes, such as zeolites (Figure 6). Nevertheless, the presence of a limited H_2_/CH_4_ selectivity impedes the possibility of achieving an extremely pure H_2_ final product, which is required in several applications (e.g., fuel cells). In fact, H_2_ purity is slightly higher than 98%, making this product suitable for internal combustion engines, injection in the grid and domestic uses, as summarized in [10]. Another important aspect to take into account is that the use of zeolite membranes requires more than a single separation stage, with the presence of a further compression unit. On the other hand, no heat exchangers have to be installed since the best selectivity values are achieved at a low temperature and embrittlement is not a problem. Figure 6 depicts the proposed plant for the treatment of an H_2_/CH_4_ equimolar mixture, consisting of three stages: concentration, H_2_ purification and CH_4_ purification. The compressed feed gas is sent to the concentration unit, which provides a retentate stream enriched in CH_4_ (up to about 93%), whereas the permeate exceeds 91.5% of H_2_ concentration. Both the streams leaving this stage are sent to a purification unit. Retentate is enriched up to 96.3% of CH_4_, while permeate can overcome 98% of H_2_. However, an additional compressor is required to restore the pressure to 20 atm before entering the H_2_ purification membrane. The retentate leaving this unit contains 94.8% of CH_4_; therefore, it is mixed with the stream exiting from the CH_4_ purification stage, which allows a final product with a CH_4_ purity of about 96%. All the details about each stream are reported in Table 5.

**Figure 7:**
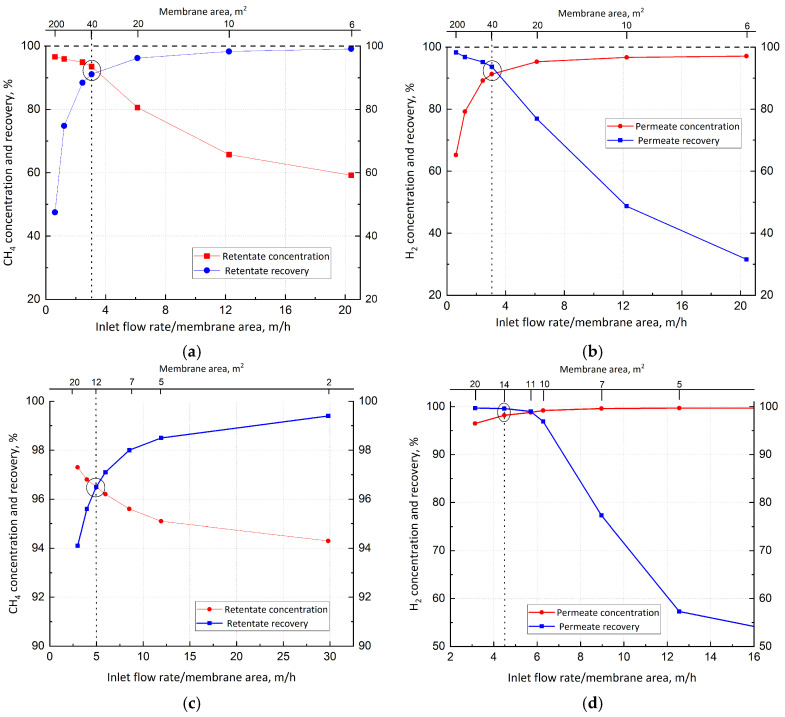
(**a**) CH_4_ concentration and recovery as functions of the inlet flow rate/membrane area ratio in the concentration unit; (**b**) H_2_ concentration and recovery as functions of the inlet flow rate/membrane area ratio in the concentration unit; (**c**) CH_4_ concentration and recovery as functions of the inlet flow rate/membrane area ratio in the purification unit; and (**d**) H_2_ concentration and recovery as functions of the inlet flow rate/membrane area ratio in the purification unit.

Figure 7 shows the dependence of concentration and recovery on the ratio between the inlet flow rate and the membrane area. The two variables show an opposite trend, resulting in an intersection point. Regarding the first membrane unit (Figure 7a,b), intersection occurs at 3.4 m/h (i.e., 36 m^2^ of area for a feed stream of 100,000 mol/h). Here, a conservative value of 3 m/h (i.e., 40 m^2^) is set, corresponding to 93.5% of CH_4_ retentate purity, with a recovery of about 91%. Similarly, H_2_ purity and recovery on the permeate side are 91.3 and 93.7%, respectively. Concerning the CH_4_ and H_2_ purification stages (Figure 7c,d), 5 and 4.5 m/h are chosen, which correspond to 12 and 14 m^2^, respectively.

**Figure 8:**
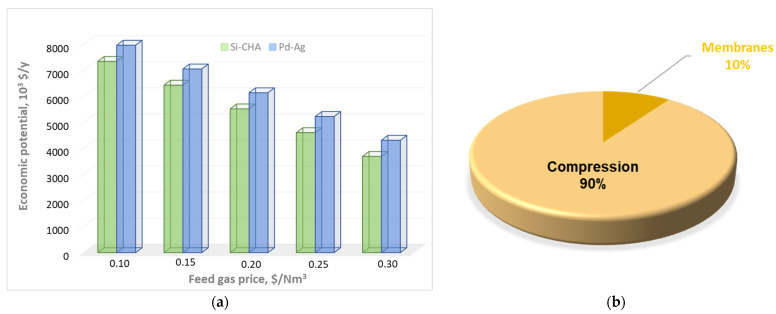
(**a**) EP as a function of the raw material price; (**b**) cost distribution of the Si-CHA configuration when raw material is excluded.

The economic potential and cost distribution of this second configuration are depicted in Figure 8. A reduction in EP compared to the previous configuration can be observed, which is attributed to the presence of a further compressor on the permeate stream leaving the first Si-CHA module. This additional cost is not compensated by savings in heating; therefore, EP shows a decrement (of about 8% at 0.1 $/Nm^3^, up to about 17% if the feed price is set to 0.30 $/Nm^3^) compared to the first scheme. In addition, cost distribution differs significantly from the previous scheme: compression increases from 67 to 90%, while membrane is reduced from 27 to 10%.

### 3.3. Purification by a Hybrid Pd–Ag/Si-CHA Membrane Plant

**Figure 9:**
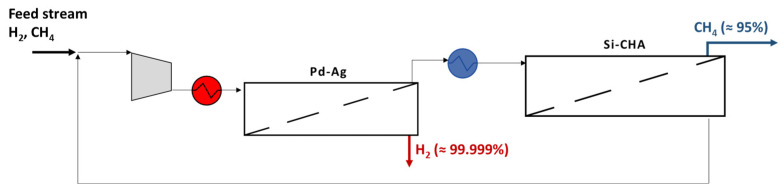
Scheme of the hybrid Pd–Ag/Si-CHA membrane plant for H_2_ and CH_4_ purification.

The hybrid configuration is chosen in an attempt to combine the advantages offered by both schemes (i.e., higher H_2_ and CH_4_ purity, respectively), while aiming to reduce the operating pressure, although it implies a greater plant complexity that makes it not practically feasible. It consists of two membrane stages: a first stage, where a Pd–Ag unit is placed to remove pure H_2_ and concentrate CH_4_; a second one, in which the Si-CHA membrane unit is able to purify the retentate stream up to about 95% of CH_4_, after a preliminary cooling back to 25 °C (Figure 9). The permeate leaving the zeolite membrane module is recycled and mixed with the feed stream to increase the recovery of both components. This configuration enables operation at a lower driving force compared to the previous ones (10 bar is set as total feed pressure) to reduce compression costs, which are the highest when the feed material is not considered. On the other hand, greater membrane areas and an additional heat exchanger are required. Regarding this scheme, 55 and 70 m^2^ are the values of the Pd–Ag and Si-CHA area (feed flow rate is 100,000 mol h^−1^), which allow achieving CH_4_ purity and H_2_ recovery of 95 and 94.7%, respectively. Table 6 reports all the details about the streams present in the plant.

**Figure 10:**
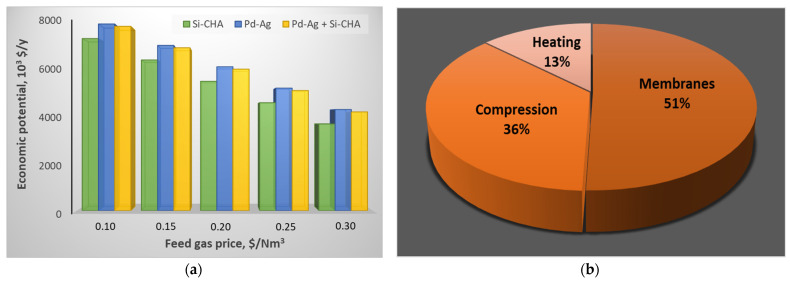
(**a**) EP as a function of the raw material price; (**b**) cost distribution of Pd–Ag/Si-CHA hybrid configuration when raw material is excluded.

However, the possibility to operate at a lower pressure does not prevail over the presence of more area, causing a slight decrement in EP compared to the single unit configuration of about 2% (Figure 10a). Therefore, the single-stage Pd–Ag solution results are the most profitable. In terms of cost distribution, the situation changes completely compared to the previous schemes. In fact, the membrane becomes the main expense (i.e., about 51%), overcoming compression (Figure 10b). In addition, 68% of the cost is given by the installation of equipment, whereas the remaining 32% is the utilities. These percentages would be completely different if the revenue due to saturated steam leaving the first heat exchanger was not considered: indeed, utilities and equipment would be 60 and 40%, respectively.

**Figure 11:**
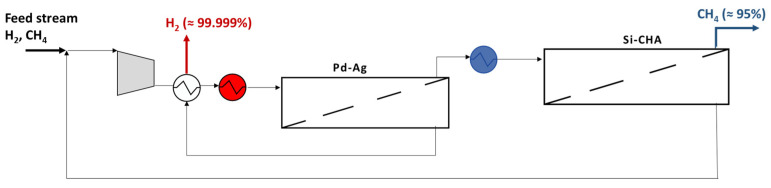
Hybrid integrated plant for gas purification.

An attempt to increase EP is carried out by exploiting the high temperature of pure H_2_ to heat the feed gas stream before entering the Pd–Ag module, as depicted in Figure 11. In this way, operating costs are reduced due to the lower demand for compressed steam. On the other hand, equipment costs increase, as an additional heat exchanger has to be installed. A further advantage of this configuration is the possibility to manage H_2_ in a safer way, since its temperature is reduced. Nevertheless, the EP increment is negligible (less than 0.10%).

A profit analysis is carried out assuming a feed gas price of 0.15 $/Nm^3^ and all the other inputs summarized in Table 7. In particular, a labor cost of 15.6 $/h is assumed, similar to what was used in a previous publication, in which it was obtained considering a monthly salary of 2500 $ [29]. Results reported in Table 7 refer to a condition in which six operators work simultaneously on the plant, assuming one worker for each operation (compression, pre-heating, heating, first membrane separation, cooling and second membrane separation). Since the plant operates 24 h per day and 7 days per week, 30 workers are supposed to be hired. The results of the hybrid scheme are compared with those of a single Pd–Ag membrane stage, considering the same labor cost. Net profit and net present value (Equations (A6) and (A7)) are estimated to be positive, indicating that both the proposed configurations could be profitable. As expected, the single-stage Pd–Ag membrane plant is more convenient.

**Figure 12:**
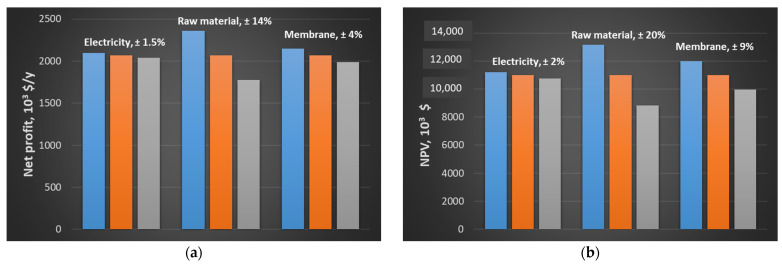
(**a**) Effect of a ±20% variation in electricity, raw material and membrane cost on net profit; (**b**) effect of a ±20% variation in electricity, raw material and membrane cost on NPV. Blue, orange and grey refer to variations of −20, 0 and +20% with respect to the set value.

A sensitivity analysis is conducted on the Pd–Ag configuration, as it proved to be the most cost-effective. In particular, net profit and NPV are estimated assuming a variation of ±20% in electricity, raw material and membrane cost. A simultaneous variation in the three parameters causes changes in net profit and NPV of about ±19 and ±31%, respectively, compared to the values reported in Table 7. On the other hand, the individual effect of each parameter is depicted in Figure 12. Raw material causes the greatest variation in net profit and NPV, being about ±14 and ±20%, respectively. Electricity price does not affect the economic performance: in fact, a variation of about 2% is observed. Membrane cost accounts for about 4 and 9%.

**Figure 13:**
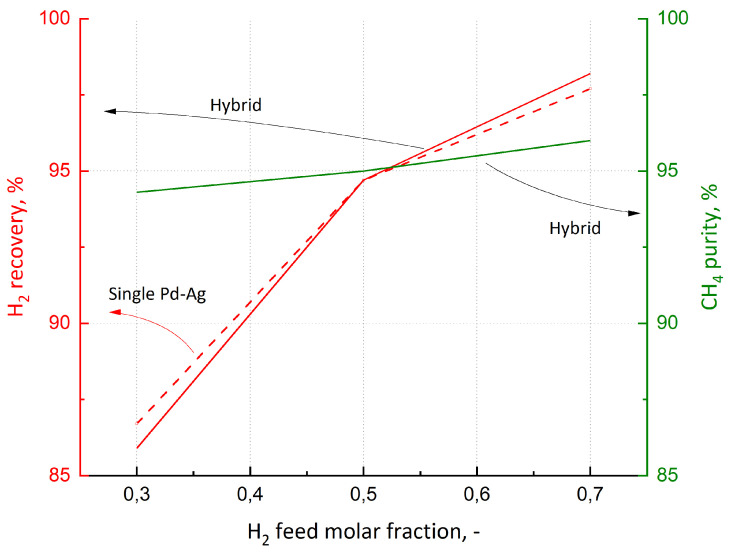
H_2_ recovery (red dashed line for Pd–Ag configuration; red solid line for Pd–Ag/Si-CHA plant) and CH_4_ purity (green line for Pd–Ag/Si-CHA configuration) as functions of the feed gas composition.

The last analysis regards the flexibility of the plants, understood as their ability to maintain purification performance despite changes in the feed composition (Figure 13). If the H_2_ concentration increased, as in the case of a coke oven gas, the performance would be better: in fact, the Pd–Ag unit would be able to recover more H_2_ as permeate product (e.g., about 98% in the case of a single Pd–Ag stage) due to the higher driving force. This would also bring a benefit to the Si-CHA unit due to the lower feed flow rate and the consequent increment of CH_4_ purity (longer residence time inside the module). On the other hand, for a feed mixture enriched in CH_4_, H_2_ recovery would be lower, leading to a more dilute CH_4_ final product. Going into detail, H_2_ recovery of the hybrid configuration oscillates between 86 and 98%, whereas CH_4_ purity changes between 94 and 96%. In the case of the Pd–Ag plant, CH_4_ concentration maintains a constant value of about 95%. These results demonstrate that both plants have good flexibility. Concerning the influence of H_2_ feed concentration on EP, the results are reported in Table 8. The lower the concentration, the greater the economic potential, due to the increment of CH_4_ flow rate produced that prevails over the reduction of H_2_ recovered. The opposite behavior is observed when H_2_ concentration increases: the decrease in CH_4_ revenue outweighs the increase in H_2_ revenue.

## 4. Conclusions and Perspectives

This work investigated the potential of membrane plants for H_2_/CH_4_ purification, showing some possible configurations that are able to achieve high purity and recovery targets. The main conclusions are listed below.

- A single Pd–Ag membrane stage can achieve ultrapure H_2_ with a recovery of about 95%, whereas all CH_4_ is recovered on the retentate with a purity of 95%. This configuration is the most cost-effective solution, having the highest EP, net profit and NPV.
- A three-stage Si-CHA configuration achieves a lower H_2_ purity of 98.3% combined with higher compression costs and lower EP.
- The hybrid Pd–Ag/Si-CHA scheme shows similar separation performance to the single Pd–Ag solution, but slightly lower EP, net profit and NPV. In addition, this configuration entails a greater process complexity compared to the single-stage Pd–Ag.
- The proposed plants are flexible: in fact, a change in feed gas concentration does not significantly affect the separation performance.

Being a preliminary techno-economic assessment, it is clear that the proposed work is based on certain simplified assumptions that facilitate its solution. The following issues have to be taken into account in a more rigorous design:

- Concerning the Pd–Ag membrane, infinite selectivity is a typical condition of single gas measurements on H_2_ and N_2_. Mixture measurements could provide finite values of selectivity and a lower H_2_ permeance, causing a reduction in purity and an increment in area.
- Regarding the zeolite membrane modules, permeance values could be affected by changes in gas concentration; therefore, in a real application, more membrane area could be necessary to realize the desired purification.
- Concentration polarization is neglected, leading to an overestimation of permeating flux. In a real application, their presence produces an increment in the required membrane area. However, a proper distribution of membrane area and recovery factor between the modules can bring concentration polarization closer to the ideal condition [47].
- Scaling up from a laboratory to a larger scale can affect the gas permeation.
- A real industrial gas mixture contains other components and impurities that influence the separation performance and require other separation equipment.

Based on these considerations, future efforts should focus on these additional factors to obtain a more realistic analysis of an industrial membrane plant.

## Figures and Tables

**Table 2:** Input parameters for simulation.

Parameter	Assumed Value
Feed flow rate, mol h^−1^	100,000
Feed composition, -	H_2_:CH_4_ = 50:50 H_2_:CH_4_ = 70:30 H_2_:CH_4_ = 30:70
Feed pressure, atm	10, 20
Permeate pressure, atm	1
H_2_ permeance, mol m^−2^ s^−1^ Pa^−1^	1.9 × 10^−3^ (Pd–Ag); 9.0 × 10^−7^ (Si-CHA)
H_2_/CH_4_ selectivity, -	Unlimited (Pd–Ag) 45 (Si-CHA)

**Table 3:** Purification targets.

Parameter	Target
H_2_ purity	>98%
CH_4_ purity	≥95%
H_2_ recovery	>90%

**Table 4:** Economic parameters used for the evaluation of EP.

Term of Equation (3)	Parameter	Assumed Value
Raw material	Feed gas price, $/Nm^3^	From 0.05 to 0.50
Onsite	Pd–Ag installation cost, $/m^2^	14,000
	Si-CHA installation cost, $/m^2^	2500
	M&S, -	2031.9 [39]
	*f_c_* compressor, -	1 [38]
	*f_m_* heat exchanger, -	1 [38]
	*f_d_* heat exchanger, -	1 [38]
	*f_p_* heat exchanger, -	0.52 [38]
Utilities	Electricity cost, $/kWh	0.10 [40,41]
	Cooling water cost, $/kg	0.000048 [42,43]
	Compressed steam cost, $/kg	0.029 [44]
Revenues	H_2_ price, $/Nm^3^	0.50 [44]
	CH_4_ price, $/Nm^3^	0.70 [45]
	Saturated steam price, $/kg	0.025 [44]

**Table 5:** Flow rate and composition of each stream present in the zeolite plant.

Si-CHA Unit	Retentate	Permeate
Concentration	Flow rate = 50,621 mol h^−1^ CH_4_ purity = 93.2%	Flow rate = 52,640 mol h^−1^ H_2_ purity = 91.6%
H_2_ purification	Flow rate = 3745 mol h^−1^ CH4 purity = 94.8%	Flow rate = 48,895 mol h^−1^ H_2_ purity = 98.3%
CH_4_ purification	Flow rate = 47,360 mol h^−1^ CH_4_ purity = 96.3%	Flow rate = 3261 mol h^−1^ H_2_ purity = 50.8%

**Table 6:** Flow rate and composition of each stream present in the hybrid plant.

Membrane Unit	Retentate	Permeate
Pd–Ag	Flow rate = 60,563 mol h^−1^ CH_4_ purity = 89.9%	Flow rate = 47,373 mol h^−1^ H_2_ purity = 99.999%
Si-CHA	Flow rate = 52,627 mol h^−1^ CH_4_ purity = 95.0%	Flow rate = 7936 mol h^−1^ H_2_ purity = 43.8%

**Table 7:** Economic inputs and results relative to the Pd–Ag and hybrid Pd–Ag/Si-CHA plants.

Parameter Type	Name	Pd–Ag	Pd–Ag + Si-CHA
Input	Feed gas price, $/Nm^3^	0.15	
	Tax index, -	0.48 [38]	
	Labor cost, $/h	15.6 [29]	
	Interest, -	0.06 [46]	
Output	Net profit, 10^3^ $/y	2069	1660
	Net present value, 10^3^ $	10,984	5552
	Internal rate of return, -	0.26	0.132

**Table 8:** EP dependence on feed composition.

Membrane Configuration	EP Variation
Pd–Ag	−10% (H_2_ feed = 70%) +10% (H_2_ feed = 30%)
Hybrid	−10% (H_2_ feed = 70%) +12% (H_2_ feed = 30%)

## Data Availability

The raw data supporting the conclusions of this article will be made available by the author on request.

## Abbreviations

The following abbreviations are used in this manuscript:

A	Area, ft^2^
CCF	Capital Charge Factor, y^−1^
EP	Economic Potential, $/y
F	Molar flow, mol/h
*f_c_*	Parameter for estimating the equipment installation cost, -
*f_d_*	Parameter for estimating the equipment installation cost, -
*f_m_*	Parameter for estimating the equipment installation cost, -
*f_p_*	Parameter for estimating the equipment installation cost, -
*hp*	Theoretical horsepower, hp
M&S	Marshall and Swift index, -
NPV	Net Present Value, $
P	Total gas pressure, Pa
Pe	Permeance, mol m^−2^ s^−1^ Pa^−n^
Tot. Prod. Cost	Total production cost, $/y
z	Axial coordinate, m
π	Mathematical constant equal to 3.14159…

## Appendix A

The Equations (A1) and (A2) are used to evaluate the onsite term [38]:

(A1) $${\;\;\;\;\;\;\;Compressor}_{Installation\;}\left(\frac{\$}{y}\right)=\;\left(\frac{M\&S}{280}\right)\left(517.5\right){\left(\frac{hp}{0.9}\right)}^{0.82}\left(2.11+f_{c}\right)\;CCF$$

(A2) $${Heat\;Exchanger}_{Installation}\left(\frac{\$}{y}\right)=\left(\frac{M\&S}{280}\right)101.3\;A^{0.65}\left(2.29+f_{c}\right)\;CCF\;\;\;$$

Installation cost of membrane is assumed to be 2.5 times the purchase cost:

(A3) $${Membrane}_{Installation}\left(\frac{\$}{y}\right)=2.5\;Purchase\;membrane\;cost\;CCF\;$$

Operating cost are calculated with the following expressions (Equations (A4) and (A5)):

(A4) $${Compressor}_{Operating}\left(\frac{\$}{y}\right)=\;\left(\frac{hp}{0.8}\right)\left(\frac{1\;kw}{1.341\;hp}\right)0.10\frac{\$}{kw\;h}\;8150\frac{h}{y}$$

(A5) $${Heat\;Exchanger}_{Operating}\left(\frac{\$}{y}\right)={Flow\;rate}_{H_{2}O}\;Utility\;\;price\frac{\$}{m^{3}}\;8150\frac{h}{y}$$

Net profit and NPV are estimated using Equations (A6) and (A7):

(A6) $$Net\;Profit=\left(1-0.48\right)\;\left[\left(Revenues-Tot.\;Prod.\;Cost\right)-Depreciation\right]$$

(A7) $$NPV=\sum_{k=1}^{t}\frac{{Cash\;Flow}_{k}}{{\left(1+j\right)}^{k}}-Initial\;investment$$

Further details are reported in [29,38].
